# New Insights into Mycotoxin Contamination, Detection, and Mitigation in Food and Feed Systems

**DOI:** 10.3390/toxins17100515

**Published:** 2025-10-20

**Authors:** Marija Kovač Tomas, Iva Jurčević Šangut

**Affiliations:** Department of Food Technology, University North, Trg dr. Žarka Dolinara 1, 48000 Koprivnica, Croatia; ijurcevic@unin.hr

**Keywords:** food and feed safety, climate change, mycotoxin contamination, innovative mitigation strategies

## Abstract

Mycotoxins are ubiquitous and unavoidable contaminants in food and feed, posing significant health risks through toxicity syndromes collectively referred to as mycotoxicoses. With climate change enhancing the conditions favorable for fungal growth and mycotoxin production, concerns over food and feed safety are increasingly pressing. Although regulatory frameworks have been established to monitor and limit the exposure, effective mitigation remains a challenge. This paper provides a comprehensive overview of recent advances in the prevention, detection, and control of mycotoxins, with particular emphasis on innovative strategies such as plant-derived bioactives, nanotechnology-based systems, genetic engineering approaches, antibody-mediated technology, and emerging non-thermal processing methods.

## 1. Introduction

Food and feed security and safety are currently facing significant challenges. Various factors, including technological advancement and socio-economic development, global trade, consequent intensification of agricultural production, and the increasingly significant impact of global climate change all influence the food and feed supply chain [1,2].

The effects of global climate change, such as the long-term changes in temperature, the amount and frequency of precipitation, and the concentration of carbon dioxide in the atmosphere, have a profound impact on the frequency and types of hazards that contaminate food [3]. Changes in the dynamics and types of pests under the influence of climate change effects lead to unexpected risks of agricultural crop contamination [4]. According to predictions, under the influence of climate change factors, pests and pathogens, including mycotoxigenic fungi and their secondary metabolites, are moving towards the poles at a rate of approximately three kilometers per year, causing geographical shifts in species distribution [5]. There are also increasing variations in interactions between crops, pathogens, and beneficial microorganisms, leading to the accelerated changes in existing contamination patterns and the creation of new plant–pathogen interactions. Additionally, indirect effects may arise from increased exposure to plant stressors, such as drought and insect damage, or changes in crop phenology, including alterations in flowering and grain maturation times [6,7].

**Figure 1:**
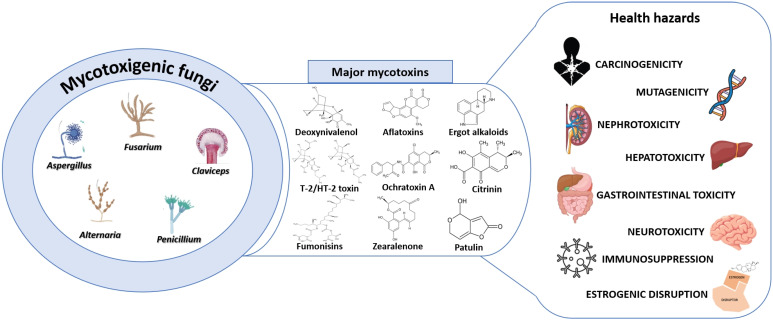
Schematic representation of the toxicological impact of major mycotoxins.

Mycotoxins, secondary metabolites produced by filamentous fungi, are widely recognized as unavoidable food and feed contaminants. They pose significant chronic dietary risks and can lead to toxicity syndromes known as mycotoxicoses upon ingestion [1,8,9]. Mycotoxins exhibit a broad range of adverse health effects, including carcinogenicity, mutagenicity, estrogenic disruption, nephrotoxicity, neurotoxicity, hepatotoxicity, immunosuppression, and gastrointestinal toxicity, with many displaying overlapping toxicities (Figure 1) [10,11,12,13].

Among them, aflatoxins, particularly aflatoxin B1, classified as a Group 1 human carcinogen, are of major concern due to their association with hepatotoxicity, immunotoxicity, mutagenicity, carcinogenicity, and teratogenicity. Ochratoxin A also possesses various detrimental effects, including nephrotoxicity, mutagenicity, carcinogenicity, immunotoxicity, teratogenicity, and neurotoxicity. Chronic exposure to trichothecenes such as T-2 and HT-2 toxins compromise immune functions, thereby increasing susceptibility to infections. Similarly, chronic exposure to deoxynivalenol, also known as vomitoxin, worsens inflammation by affecting gastrointestinal epithelial cells and immune mechanisms. Zearalenone functions as an endocrine disruptor by binding to estrogen receptors, resulting in hormonal imbalance and reproductive disfunction. The toxicity of fumonisins, primarily B1 and B2, is very much species-dependent, with potential to cause neurotoxicity, hepatotoxicity, nephrotoxicity, and mammalian cytotoxicity. Ergot alkaloids possess neurotoxic properties and reduce productivity in animals, often linked to gastrointestinal symptoms and internal bleeding [10,11,14,15]. The exposure to mixtures of different mycotoxins is another toxicological concern due to possible additive and synergistic effects that are yet to be fully characterized [8].

Given their widespread presence, global mycotoxin surveys indicate a worldwide crop contamination rate of up to 60–80%, with *Fusarium* mycotoxins being most dominant in temperate climates [1,6,8,16]. To mitigate risks associated with mycotoxin exposure, regulatory authorities have established threshold limits for major mycotoxins. Within the European Union (EU), maximum permitted or guidance levels in various food- and feedstuff are set by the Commission Regulation 2023/915 [17], Commission Recommendation 2006/576/EC [18], and Directive 2002/32/EC [19], respectively. Detailed limits, along with corresponding values from the United States (US) Food and Drug Administration (FDA) for broader perspective, are summarized in Table 1. Despite these regulatory measures, the complete prevention of mycotoxin contamination remains challenging, particularly in the light of climate change effects, which heightens the concerns regarding food and feed safety. In response, ongoing research and the development of comprehensive mycotoxin management approaches are essential throughout food and feed chain. Beyond the conventional approaches, increasing attention is being directed toward innovative methods, such as the use of botanicals, nanotechnology, genetic engineering, antibody-mediated technology, and novel non-thermal processing techniques [20,21,22,23]. Therefore, this review aims to provide an in-depth overview of methods for mycotoxin prevention, detection, and control, with an emphasis on recent advances and effective strategies to enhance safety across the agri-food chain.

## 2. Mycotoxin Contamination and Mitigation Strategies

**Figure 2:**
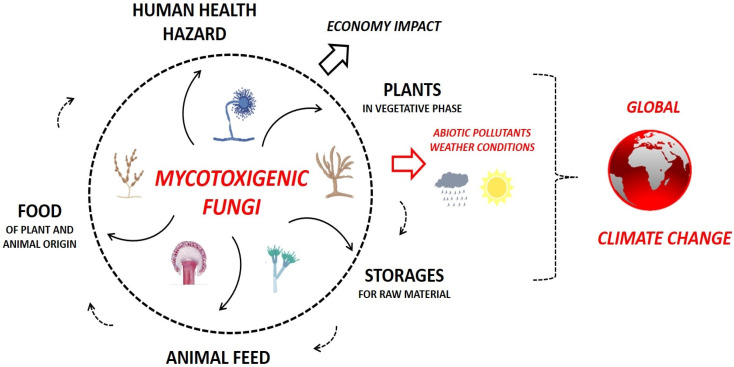
Scheme of mycotoxin contamination cycle and its influencing factors, adapted from [1].

Mycotoxin contamination can occur at any point in the food and feed production and supply chain (Figure 2): in the fields, during crop growth; and afterwards, during crop manipulation, storage, and processing [1,6].

The most relevant mycotoxigenic fungi belong to the *Aspergillus*, *Fusarium*, *Alternaria*, *Penicillium*, and *Claviceps* genera, each exhibiting specific environmental preferences for growth and development. Generally, *Aspergillus* species thrive under conditions of low water activity and elevated temperatures, whereas *Fusarium* species favor higher water activity and moderate temperature ranges [6,9,25,26]. Mycotoxin biosynthesis, and, consequently, their occurrence and contamination, is therefore strongly influenced by environmental factors, including rainfall, air temperature, relative humidity, and substrate characteristics. Additionally, the physiological state of the fungus and its interactions with other microorganisms also have an important role [6,10,27]. Although optimal conditions vary between fungal species, many toxigenic fungi grow well within a temperature range of 10–40 °C, at a pH value around 8.4, and water activity levels above 0.70. Moreover, some fungal species are capable of producing multiple mycotoxins, while certain mycotoxins can be synthesized by more than one fungal genus. Consequently, the occurrence of several mycotoxins in a single contaminated sample is not uncommon [6,10]. Given the complex interplay of environmental and biological factors affecting fungal growth and mycotoxin production, understanding the regional patterns of mycotoxin occurrence is essential for accurate risk assessment, as well as the implementation of effective mitigation strategies.

### 2.1. Mycotoxin Occurrence and Detection

#### 2.1.1. Mycotoxin Occurrence in Food and Feed

The climate of Croatia, positioned in the northern temperate zone, has experienced significant temperature and precipitation changes over the last 20 years, driven by climate change. Trends toward warmer and drier conditions have been recorded, impacting agricultural productivity, human well-being, and entire ecosystems [16], mirroring broader patterns across temperate Europe. This regional climate variability provides an important context for understanding mycotoxin occurrence under evolving environmental conditions.

Mycotoxin surveys in Croatia have frequently detected *Fusarium* mycotoxins as the primary contaminants of cereals [6,25,28,29], with occasional reports of aflatoxin dominance [30,31,32]. A study on the presence of 11 EU-regulated mycotoxins in cereals from seasons 2016 and 2017 revealed DON as the most prevalent mycotoxin, detected in 73% of samples from 2016 and 33% from 2017. Maize was the most contaminated cereal crop type, with FUM as the most abundant mycotoxin. The co-occurrence of multiple mycotoxins was common, especially *Fusarium* mycotoxin combinations, and mycotoxin presence and concentrations were correlated with meteorological conditions during plant flowering, causing more severe contamination in 2016 [6]. Similarly, an analysis of various cereal samples, including maize, wheat, triticale, oats, and barley from the 2016/2017 harvest years, showed that *Fusarium* metabolites contaminated over 50% of the samples [25]. Additionally, a study of major mycotoxin (AFB1, OTA, ZEN, DON, FUM) occurrence in unprocessed cereals and cereal-based products from conventional and organic production in 2015 revealed no significantly different mycotoxin contamination between organic and conventional samples, highlighting weather conditions, cultivation year, location, crop rotation, and tillage as the main contributors to mycotoxin synthesis rather than the type of production system [29].

AFB1 prevalence is generally observed in maize and maize-based feed, strongly influenced by climatic conditions. A study of AFB1 occurrence in maize during the 2018–2021 period showed the highest AFB1 prevalence in 2021, with contamination levels up to 40%, largely attributed to hot and dry weather conditions. In contrast, the period 2018–2020 showed much lower contamination rates at only 20% [31]. A five-year monitoring study (2009–2013) of AFB1 monitoring in grains and feed from Croatian dairy farms revealed significantly higher concentrations of AFB1 in maize, grain mixtures and dairy cattle feed collected during 2013, linked to extreme warm and drought weather conditions during the crop growth and harvesting in 2012. Regional variations were also observed, with significantly higher AFB1 levels in samples from eastern Croatia [30,32].

Elevated mycotoxin levels in agri-food commodities consequently heighten the risk of mycotoxin carry-over along the food production chain, affecting animal-derived foods, including milk, dairy products, and traditional dry-cured meat products [16]. Notably, the presence of AFM1 in milk increased after 2012 in response to the mentioned increased AFB1 maize contamination, peaking in 2013 when AFM1 exceeded EU threshold limits in 28% and 10% of raw and UHT milk samples, respectively [33]. Subsequent years showed reduced AFM1 contamination due to more favorable weather conditions and improved feed management, while sporadic elevated concentrations were linked to localized contaminated feed use and a pronounced seasonal pattern, with higher contamination levels observed in autumn and winter months [34,35,36].

Given its importance as a dietary protein source, the mycotoxin presence in meat products raises additional food safety concerns. Mycotoxins frequently associated with meat include OTA and AB1, with recent studies also investigating CIT, cyclopiazonic acid (CPA), and sterigmatocystin (STC), a biosynthetic precursor of AFB1. Their occurrence in meat products can result from several factors: direct contamination through fungal growth on product surface, indirect contamination via the use of contaminated spices, and the aforementioned carry-over effect, i.e., transfer from contaminated feed in farm animal diets [16,37]. Although not all of the details of mycotoxin transfer in farm animals have yet been fully elucidated, research has demonstrated higher OTA transfer rate to liver and kidney tissue, and also AFB1 carry-over to pig liver, muscle, and fat tissue [16,38,39]. Additionally, a recent study on CIT reported its potential transfer from animal feed into edible pig tissues, although with low carry-over rates [40]. A Croatian study targeting five relevant mycotoxins (OTA, AFB1, CIT, CPA, and STC) in traditional dry-cured meat products found 27% of contaminated samples, with CPA and OTA being the most frequently detected. A notably higher contamination rate was observed in dry-fermented sausages compared to other dry-cured meat products, underscoring the need for further research into all contamination pathways and the implementation of preventive measures across all stages of meat production and storage [41].

#### 2.1.2. Mycotoxin Detection Techniques

There are various methods and techniques employed for mycotoxin determination. In general, mycotoxin analysis is a complex process that requires appropriate sampling strategies, sample preparation procedures, and reliable detection and quantification using suitable analytical instruments. Formerly, the enzyme-linked immunosorbent assay (ELISA) was the predominant method in mycotoxin analysis; however, liquid chromatography coupled to (tandem) mass spectrometry (LC-MS/MS) is now more commonly employed. Given the frequent mycotoxin co-occurrence, as previously mentioned, LC-MS/MS-based multi-mycotoxin methods enable the simultaneous and unambiguous determination of a wide range of chemically diverse compounds within a relatively short analysis time [9]. Since the first reported multi-mycotoxin method in 2006 [42], several LC-MS/MS methods have been developed for both free and modified mycotoxin forms, legislatively regulated or unregulated, in a variety of food and feed matrices [43,44,45,46,47,48]. Despite its widespread use, LC-MS/MS has certain limitations, including a restricted number of analytes that can be measured in a single run due to instrument capacity; the need for analytical standards for compound identification and quantification; susceptibility to matrix effects that may affect accuracy; and its inherent focus on targeted analysis only [9]. To overcome these limitations and to complement the LC-MS/MS methods, both targeted and non-targeted high-resolution mass spectrometry (HRMS) methods have been employed in mycotoxin research, especially in more obscure crops and feed [49,50,51,52].

In addition to the established laboratory-based methods, approaches such as on-site detection techniques and biosensor technologies are promising tools for rapid, cost-effective, and portable mycotoxin screening. These methods aim to enhance real-time monitoring and early risk assessment in food and feed production chains. Among these, immune-based lateral flow assay (LFA) is a widely used diagnostic tool allowing qualitative and quantitative analyte determination, offering shorter detection times and simpler execution compared to the abovementioned ELISA. Although limitations such as susceptibility to false positive results caused by matrix interferences or reproducibility and sensitivity issues exist, recent advancements have addressed these challenges [15,53]. For instance, polydopamine-coated gold nanoparticles were developed to enhance method sensitivity, as demonstrated for ZEN detection in maize [54]. Furthermore, multiplex LFA has been reported, allowing for the simultaneous detection of multiple mycotoxins within a single test [55], while aptamer-based LFA has shown superior specificity and lower detection limit, for instance, 0.1 ng/mL for AFB1 [56]. Owing to its portability, simplicity, and rapid results, LFA is particularly suitable for field-level mycotoxin analysis as a one-step screening tool [15,22].

Recently, more sophisticated detection systems have emerged. To differentiate contaminated from uncontaminated commodities, various optical imaging techniques, such as color, fluorescence, infrared, or hyperspectral imaging systems (HISs), have been employed. These methods aim to detect either the presence of mycotoxins or changes in the characteristics of commodities associated with fungal growth. New artificial intelligence and machine learning approaches have been integrated into mycotoxin detection flows to enhance the classification of samples as acceptable or unacceptable (i.e., below or above regulatory thresholds) and to predict toxin concentration using statistical models or algorithms that support decision-making [49]. Several prediction models have been specifically developed for the evaluation of mycotoxins in cereals—for example, DON in wheat [57], T-2 and HT-2 in oats [58], and FUM and AFT in maize [59]—as well as for the quantitative prediction of AFT in peanuts [60]. Furthermore, the integration of biosensors with digital technologies enables real-time alerts upon mycotoxin detection. In general, biosensors are analytical tools that use a biological recognition element, such as enzymes, antibodies, or nucleic acids, coupled with a signal transducer to detect and quantify specific compounds. The application of deep learning algorithms enhances the interpretation of biosensor output data by processing complex signal patterns and improving detection accuracy, particularly in distinguishing between different types of contamination [15,49].

### 2.2. Mycotoxin Mitigation Strategies

At the regulatory level, the European Union monitors food and feed contamination using the Rapid Alert System for Feed and Food (RASFF), enabling timely communication among member states regarding potential hazards. The most recent European Commission report analyzing data exchanged through the electronic iRASFF system in 2024 identifies mycotoxins, particularly AFT and OTA, as one of the most frequently reported hazards. The food and feed categories most commonly linked to mycotoxin contamination include nuts and nut products, fruits and vegetables, and cereals and bakery products [61]. These regulatory findings underscore the importance of implementing control measures throughout the food and feed production and supply chain. The pre-harvest measures focus on preventing the initial contamination of crops in the field by mycotoxigenic fungi, thereby minimizing the risk of mycotoxin formation. Nevertheless, if contamination does occur, various post-harvest measures can be applied to reduce mycotoxin concentration and mitigate their potential adverse effects [10,23,62,63].

Pre-harvest measures primarily include agronomic strategies such as crop rotation, proper soil cultivation, balanced fertilization, the use of appropriate seed material and sowing techniques, crop breeding and selection, and seed priming—a set of techniques that prepare plants for potential abiotic and biotic stressors [10,23,62,63,64]. Post-harvest measures refer to various physical, chemical, or biological methods for mycotoxin decontamination and detoxification, with the latter being regarded as more specialized, effective, and environmentally friendly [23,62].

#### 2.2.1. Physical Treatment

The cleaning and sorting of agricultural commodities is undoubtedly the first step in the natural mitigation of mycotoxins. Mycotoxins, particularly AFT, are known to be heterogeneously distributed within a batch, with the highest concentrations typically found in damaged or discolored kernels [63,65]. This heterogeneous distribution underscores the importance of proper sampling procedures [49]. Sorting, whether performed manually or using sorting machines, is essential for effective mycotoxin reduction [63]. Recent research has advanced beyond traditional sorting approaches relying on external indicators of fungal presence such as grain size, shape, and color toward technologies for direct mycotoxin detection. Fluorescence-based sorting offers enhanced capabilities for grain analysis [63,66]. Its commercialization has been accelerated by the integration of innovative tools such as the previously mentioned HIS approach, which adds a spatial dimension to conventional spectroscopic techniques, thus enabling the mapping of chemical components within a sample [66]. This commercially available sorting system, employing LED light and HIS, is capable of processing 15 tons of maize per hour, achieving an AFT reduction of 85–90% in validation trials. In addition, laser-induced fluorescence-based sorters have also recently been employed for real-time AFT detection in maize [67,68].

Managing mycotoxin contamination extends to critical post-harvest storage conditions, particularly elevated temperature and high relative humidity, as they directly influence fungal growth and secondary metabolite production. Inadequate storage practices continue to contribute to post-harvest losses in many developing regions, with crop losses of 20–50%. Effective storage management, including controlled air temperature (<20 °C), relative humidity (<80%), and proper ventilation, has been shown to inhibit fungal development and limit mycotoxin synthesis [10,66].

During cereal processing, mycotoxins are often reduced in fractions intended for human consumption, but may be concentrated in by-products frequently used as animal feed, depending on the applied processing method [63,65]. Not all parts of the grain kernel are equally susceptible to fungal contamination: the outer parts such as the pericarp and germ are more prone to contamination than the endosperm, and therefore typically contain higher mycotoxin concentrations. For instance, grain milling has been shown to redistribute mycotoxins into fractions such as bran, feed flour, and polishing, highlighting the need for targeted mycotoxin mitigation strategies at various stages of processing [10,69,70].

Time and temperature combination are some of the crucial factors in industrial processing that affect mycotoxin concentration in the final product. However, most mycotoxins are thermally stable under conventional temperature regimes up to 100 °C, while higher temperatures applied in frying, roasting, toasting, or extrusion might result in mycotoxin reduction [69,71]. For instance, AFM1 remains resistant to commonly used thermal processing treatments in the dairy industry, such as pasteurization and ultra-high-temperature processing [16]. On the other hand, the roasting of coffee beans has demonstrated a decrease in OTA content by up to 97%, depending on factors such as roasting and particle size [72], with thermal degradation products being less toxic than the parent compound [73]. Similar effects have been observed for AFT, with reductions of 50–70% in peanuts and pecans and of 40–80% in maize, depending on the initial mycotoxin concentration and type and temperature of the process [74]. The extent of mycotoxin reduction during extrusion has been found to largely depend on process variables, including temperature, screw speed, feed moisture content, and residence time. Under optimized conditions, it has been reported to significantly reduce or even completely eliminate certain mycotoxins [75]. In the case of FUM, the presence of certain additives, such as reducing sugars and sodium chloride, is especially important. Temperatures at 160 °C and above, in combination with glucose, have shown the greatest FUM degradation [9,76]. For example, extrusion with glucose more effectively reduced FB1 concentrations of the maize grits (75–85%) than extrusion alone (10–28%), with reduced toxicity also being observed [76]. This effect is likely due to Maillard-type reactions, in which FB1 reacts with reducing sugars to form degradation products such as N-carboxymethyl FB1 and N-deoxyfructosyl FB1 [9,77].

Other physical treatments, including emerging techniques such as cold plasma, gamma radiation, or microwave heating, have been recognized as a valuable tool in mycotoxin mitigation, and therefore will be addressed in Section 3.1.

#### 2.2.2. Chemical Control

Chemical agents used for mycotoxin decontamination can be sorted into several categories, such as alkaline compounds (ammonia, sodium hydroxide, calcium hydroxide), acids (acetic acid, phosphoric acid, formic acid, propionic acid, sorbic acid, sodium hypochlorite), reducing agents (sodium bisulphite, sugars), oxidizing agents (ozone, hydrogen peroxide), and chlorinating agents. While chemical treatments can be effective in mycotoxin decontamination, there are certain limitations regarding their safety and the deterioration of treated samples [10,69]. Commission Regulation (EU) 2023/915 currently prohibits the chemical detoxification treatment of food for human consumption [17].

The effectiveness of chemical methods varies in terms of both of mycotoxin and matrix type. For instance, the ammoniation method was proven successful for AFB1 decontamination in animal feed, largely due to its instability under alkaline conditions. On the other hand, it is advised to avoid the alkaline treatment of FUM-contaminated samples until full knowledge has been obtained on the toxicity of their hydrolyzed products among various species [14,71]. Calcium hydroxide has shown potential in T-2 and diacetoxyscirpenol decontamination in feedstuff, as their structures can be modified under alkaline conditions [69]. Similarly, alkaline solutions of sodium hydroxide and potassium hydroxide have been employed for AFB1 degradation in contaminated oil. These chemicals can, however, cause secondary contamination and impair the nutritional composition of the products [78]. The biological activity of AFB1 can also be diminished by applying strong acids. Treatment with hydrochloric acid has been shown to reduce AFB1 concentration by 19.3% within 24 h. Certain OTA degradation methods using formic, propionic, and sorbic acids has been recorded (0.25–1.0%) after exposure for 24 h [69]. Reducing agents have been proven to react with AFT and trichothecenes. The treatment of cereal grains and animal feed with sodium bisulphite and metabisulfite to reduce DON contamination has been demonstrated in several studies, revealing the formation of less toxic DON sulfonate [71,79]. Moreover, oxidizing agents such as hydrogen peroxide have proven useful in mycotoxin degradation [80]. For example, dried fig treatment using a 0.2% aqueous solution of hydrogen peroxide prior to sodium bisulphite application resulted in 66% AFT degradation in 72 h [81]. Maize treated with 10% hydrogen peroxide at 80 °C for 16 h showed an 84% reduction in ZEN content [82]. More recently, hydrogen peroxide has been combined with a novel magnetic palladium-based composite for the efficient reduction of AFB1 in edible oil. Using 4 mM hydrogen peroxide and 0.1 mg/mL of the composite, over 94% of AFB1 was degraded within 60 min across a wide pH range, without affecting the physicochemical properties of the oil, thereby offering a promising detoxification strategy using a stable, well-dispersed catalyst system [83].

Among chemical methods, natural alternatives such as chitosan or ozone treatments have gained attention as a safer option for mycotoxin control. A natural polysaccharide chitosan, with designated antimicrobial characteristics, has been investigated for *Fusarium* species (*F. proliferatum*, *F. verticillioides*, and *F. graminearum*) control in maize and wheat. It has been demonstrated that low-molecular-weight chitosan with more than 70% deacetylation was able to reduce DON and FUM production at the lowest applied dose of 0.5 mg/kg. Chitosan has therefore been characterized as an important alternative for mycotoxin mitigation pre- and post-harvest [84]. Another study, employing brown marine algae *Ascophyllum nodosum* in combination with chitosan in the treatment of Fusarium Head Blight disease caused by *Fusarium graminearum*, demonstrated DON contamination reduction in wheat grains [85]. In parallel, ozone has emerged as a powerful, residue-free treatment for microbial and mycotoxin decontamination, often applied in food storage, for sterilization and mycotoxin control. As a strong oxidizing agent, ozone decomposes and destroys microorganisms and organic matter in water, leaving toxic products [14,86]. The efficiency of gaseous ozone was proven in reducing DON and its glucoside form DON-3-glucoside, bacteria, fungi, and yeasts in naturally contaminated durum wheat. Under optimal conditions (55 g O_3_ h^−1^ for 6 h), toxins were reduced by 29 and 44%, respectively, while no changes were observed in the chemical and rheological properties of wheat and its products. Ozonation also produced a significant reduction in the total count (CFU/g) of bacteria, fungi, and yeasts in wheat grains [87]. Another study demonstrated a reduction in DON-contaminated wheat toxicity after short-time ozone treatment, while there was no observed effect on the quality of wheat [88]. Reactive oxygen species have also been suggested for degrading AFB1 and AFG1 due to their susceptible chemical structure [89].

#### 2.2.3. Biological Control

Biological detoxification is often regarded as a more environmentally friendly and convenient alternative to physical and chemical approaches. It encompasses methods for mycotoxin detoxification using biological agents, microorganisms, and their enzymes through processes of biosorption and biodegradation [22,90].

Lactic acid bacteria (LAC), commonly used in fermented food production, are found to have an excellent binding capability with AFT, namely, AFB1 and AFM1 [22,91]. For instance, *Lactobacillus rhamnosus* was found to significantly bind with AFB1 from wheat flour during bread-making processes [92]. Moreover, probiotic cultures, primarily *Lactobacillus casei* LC-01, but also yoghurt culture YC-380, showed high efficiency in the reduction of AFM1 concentration during fermented milk production [93]. Probiotic preparation containing LAB bacteria, *Saccharomyces cerevisiae* yeasts, and *Yucca schidigera* extract decreased AFB1 during the fermentation of feed mixture [94]. Furthermore, mycotoxin binders composed of baker yeast (*Saccharomyces cerevisiae*), bentonite, and silymarin ameliorated aflatoxicosis effects in broilers [95]. Distillery yeast sludge, containing *Saccharomyces cerevisiae*, *Candida parapsilosis*, and *C. guilliermondii*, used as a feed additive in poultry industry, was found to be very efficient against OTA- and AFB1-induced immunosuppression [96]. In addition to the use of microorganisms, various mycotoxin binders have been applied to reduce toxin bioavailability in animal feed. These include inorganic materials such as bentonite, zeolite, and activated charcoal, as well as plant fibers. Acting primarily through adsorption in the gastrointestinal tract, they limit toxin adsorption and promote their excretion [22].

Apart from binding mycotoxins, some microorganisms, including bacteria, fungi, and yeasts, also have the ability to detoxify mycotoxins into less toxic products through processes of acetylation, glycosylation, ring cleavage, hydrolysis, deamination, or decarboxylation [22]. For example, *Lysinibacillus* sp. ZJ-2016-1, isolated from chicken large intestines, showed excellent ability in ZEN reduction [97], while *Eggerthella* sp. DII-9 was found to be capable of trichothecene mycotoxin de-epoxidation with high efficiency [98].

Certain non-toxigenic fungal strains exhibit potential for mycotoxin control. In particular, non-toxigenic *Aspergillus flavus* was found to be a good competitor against mycotoxigenic *A. flavus*, thereby potentially reducing AFT contamination, and also engaging plant defense mechanisms [90]. The treatment of soil with competitive non-toxigenic fungi strains has been shown to protect crops during growth and storage by competing for infection sites [23,99].

Enzymes purified from microbial systems, such as oxidase, peroxidase, laccase, reductase, esterase, carboxylesterase transaminase, or lactose hydrolase, have been proven to degrade mycotoxins both in vivo and in vitro. The application of enzymes is considered safe and easy, ensuring homogeneity and reproducibility, compared to the application of microorganisms [22,90]. Peroxidase enzymes from *Aspergillus* species and horseradish peroxidase from *Raphanus sativus* have been proven to exhibit AFB1 degradation activity [100]. The effective enzymatic degradation of AFB1 has also been performed by extracellular extracts of *Rhodococcus erythropolis* along with laccase enzymes from several fungal species [101]. Carboxypeptidase enzymes from *Brevibacterium* species and *Bacillus subtilis* were reported to degrade OTA [102,103]. The epoxidase enzyme from *Eubacterium* BBSH 797 was reported to detoxify DON to its de-epoxy form [104]. FB1 detoxification was proven to be at least two enzymatic steps, comprising de-esterification and deamination, resulting in hydrolyzed FB1 [105].

Despite the wide range of physical, chemical, and biological methods available for mycotoxin mitigation, effectively neutralizing these compounds remains a significant challenge. The high stability of many mycotoxins, their diverse behavior across different food and feed matrices, and concerns regarding the toxicity and safety of their degradation products complicate efforts to achieve complete safety. These factors underscore the ongoing need for continued research into innovative strategies, paving the way for more effective and sustainable mycotoxin control.

## 3. Innovative Approaches for Mycotoxin Mitigation

**Figure 3:**
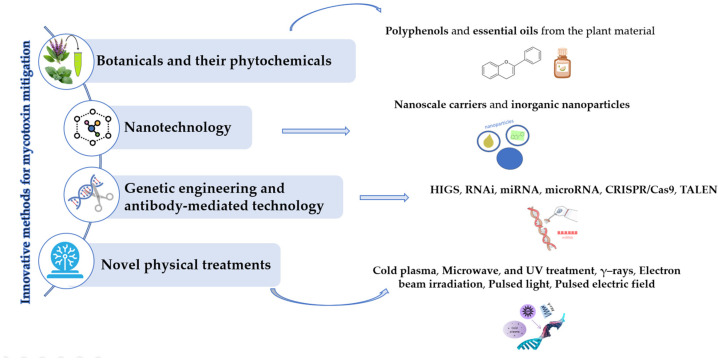
Schematic overview of current innovative methods for mycotoxin mitigation.

In addition to conventional methods for mycotoxin control, a variety of innovative approaches have gained increasing attention due to their potential to effectively reduce mycotoxin contamination, without leaving toxic residues or compromising product quality. These novel and promising strategies, which are still under development, include the use of botanicals and phytochemicals, nanotechnology, genetic engineering and antibody-mediated technology, as well as emerging non-thermal processing (Figure 3) [20,21].

### 3.1. Botanicals and Their Phytochemicals

Plants are an excellent source of specialized metabolites, i.e., phytochemicals that contribute to defense against various environmental stressors. Key representatives include polyphenolic compounds such as flavonoids and phenolic acids, terpenoids, phytosterols, alkaloids, and essential oils [106]. Polyphenols have recently gained attention for their potential role in controlling mycotoxins in food systems due to their diverse biological activities, including antiviral, antimicrobial, antioxidant, and anti-inflammatory effects [21]. Among these, antioxidant capacity and lipophilicity appear to play a crucial role in their antimycotoxigenic activity. Several mechanisms have been proposed to elucidate mycotoxin inhibition, including the structural disruption of the fungal membrane, downregulation of genes involved in mycotoxin biosynthesis, and inhibition of oxidative stress-regulating enzymes [107]. Multiple studies have investigated the application of specific plant extracts and polyphenolic compounds, demonstrating their efficacy in reducing fungal growth and mycotoxin synthesis. For instance, aqueous extracts from *Salvia farinacea* and *Azadirachta indica*, along with polyphenolic compounds, have been shown to inhibit the development of several *Aspergillus* species on various meat products, significantly reducing AFT and OTA production [108]. Phenolic extracts also inhibited AFB1 formation in edible beans, with chlorogenic and gallic acid identified as key protective compounds [109]. Furthermore, flavones derived from citrus residues have demonstrated the inhibition of PAT production from *Penicillium expansum*, *Aspergillus terreus*, and *Byssochlamys fulva* by at least 95% [110]. More recently, cyclodextrin-based nanosponges encapsulating plant extracts or bioactive compounds have been proposed as innovative and environmentally friendly tools for mycotoxin decontamination [106]. These findings highlight the promising potential of botanicals for integration into future food and feed safety and mycotoxin mitigation strategies.

In addition to plant extracts and polyphenols, natural essential oils have been extensively studied for their antifungal and antimycotoxigenic properties. Their mechanisms of action include enzyme inhibition (involved in carbohydrate degradation and mycotoxin biosynthesis) and the disruption of fungal cell membrane integrity [15,21]. For instance, *Mentha spicata* essential oil demonstrated antifungal and antiaflatoxigenic efficacy against a toxigenic strain of *Aspergillus flavus* in chickpea food systems during storage [111]. Similarly, turmeric (*Curcuma longa* L.) essential oil showed strong antifungal activity against *A. flavus*, reducing AFT infestation in maize [112]. Despite their promising potential in mycotoxin mitigation, natural essential oils are associated with several challenges such as variable efficacy, sensory impact, regulatory gaps, safety concerns, and practical limitations [15]. New findings indicate that encapsulation considerably enhances the performance of certain essential oils. For instance, *Origanum majorana* L. essential oil encapsulated in a chitosan nanoemulsion has recently been reported as a novel preservative for stored foods, effectively inhibiting fungal growth, AFB1 production, and lipid peroxidation without altering their sensory attributes, while also presenting a favorable safety profile [113].

Nonetheless, the broader practical application of phytochemicals in food systems remains constrained due to their low chemical stability and limited bioavailability, as well as the lack of standardized protocols for their formulation and use [114].

### 3.2. Nanotechnology

A promising approach in nanotechnology-based mycotoxin control involves the development of nano-delivery systems for the targeted application of natural antimycotoxigenic agents. As discussed in the previous section, these nanofungicide formulations typically encapsulate essential oils and plant-derived bioactive compounds, including flavonoids, alkaloids, and other phytochemicals, into nanoscale carriers to enhance their stability, solubility, and controlled release, ultimately improving their efficacy against mycotoxigenic fungi and other pathogens [21,22]. Given that plants lack circulating immune cells to destroy pathogens, but only rudimentary immune responses, the application of such green nanofungicides represents a novel strategy to enhance plant defenses and prevent fungal infections [115]. Moreover, polymeric nanofungicides have been proposed for dual-function agriculture use. For example, the application of resveratrol-loaded chitosan nanoparticles offers a dual purpose: the antifungal activity of resveratrol mitigates fungal proliferation and subsequent mycotoxin synthesis, while chitosan contributes to mycotoxin decontamination through its high adsorption capacity [116]. In addition to encapsulating natural products, converting conventional chemical fungicides into nanoforms has also demonstrated improved performance. Notably, the commercial fungicides trifloxystrobin and tebuconazole exhibited improved fungicidal activity against the plant pathogen *Macrophomina phaseolina* after being processed into nanoform using the ball milling method [117].

In parallel, recent research has increasingly explored the use of inorganic nanoparticles as active agents with direct antifungal and antimycotoxigenic properties. Their mechanisms of action in mitigating mycotoxin-related risks are associated with their ability to modulate oxidative stress, trigger inflammatory responses, or interact with nucleic acids, thereby contributing to the destruction of microorganisms in both higher plants and animals [21]. Fullerol C_60_(OH)_24_ nanoparticles (FNP), for instance, have been shown to alter the secondary metabolism of several important foodborne mycotoxigenic fungi, including species from the *Aspergillus*, *Fusarium*, *Alternaria*, and *Penicillium* genera. In this study, fungal cultures were grown in liquid RPMI 1640 media for 72 h at 29 °C, and secondary metabolites were analyzed by the LC-MS/MS dilute and shoot multi-mycotoxin method. Exposure to FNP (at concentrations of 1–1000 ng/mL) resulted in a reduction of 35 fungal secondary metabolite concentrations, depending on the applied FNP dosage and fungal genus. These findings contribute to a better understanding of the real nature of FNP–fungus interactions and their potential implications for food safety [26]. Similarly, silver nanoparticles (AgNP) (14–100 nm) have demonstrated antifungal activity against major toxigenic *Fusarium* species. Their efficacy was shown to depend significantly on fungal species, nanoparticle concentration (in the range of 2–45 μg/mL), exposure time (2–30 h), and their interactions, affecting spore viability, lag period, and growth rate in subsequent cultures grown in maize-based medium. Notably, none of the treatments led to increased conidial germination, growth, or mycotoxin biosynthesis with respect to controls. These findings support the antifungal potential of AgNP and suggest its applicability as a novel active ingredient in bioactive polymers (paints, films, or coatings) for use in the agri-food sector [118].

Together, these advancements in nanotechnology highlight its diverse and promising role in mycotoxin mitigation, offering both enhanced delivery systems for natural compounds and the direct use of nanomaterials with antifungal properties.

### 3.3. Genetic Engineering and Antibody-Mediated Technology

Mycotoxin contamination at both pre- and post-harvest can be significantly mitigated through developing disease-resistant traits using advanced biotechnological approaches [22]. Transgenic techniques such as host-induced gene silencing (HIGS), RNA interference (RNAi), microRNA (miRNA)- or artificial microRNA (amiRNA)-mediated gene silencing, designer transcription activator-like effector (dTALE)-mediated regulation of gene expression, Zn-Finger nucleases, mega-nucleases, transcription activator-like effector nucleases (TALEN), clustered regularly interspaced short palindromic repeats (CRISPR/Cas9), and oligonucleotide-directed mutagenesis (ODN)-based gene-editing techniques have shown promise in enhancing resistance to mycotoxin-producing fungi. In particular, the HIGS strategy allows the host plant to silence fungal genes without expressing foreign proteins [22,119]. Similarly, the RNAi approach demonstrated success in silencing fungal genes essential for mycotoxin biosynthesis. For instance, transgenic peanut plants with RNAi targeting five AFT biosynthesis genes showed a 100% reduction in AFB1 and AFB2 upon infection with aflatoxigenic *Aspergillus flavus* [120]. Furthermore, the dual silencing of Bc-Dcl1 and Bc-Dcl2 genes in *Botrytis cinerea* significantly reduced fungal virulence, highlighting their potential as targets for broad-spectrum resistance [121]. The role of microRNAs in plant disease resistance has also been explored. Overexpressing the Osa-miR7696 gene in transgenic rice plants has been shown to enhance resistance against *Magnaporthe oryzae* [122]. Moreover, overexpressing the barley antifungal gene HvNEP-1 reduced Fusarium head blight disease severity and progression after inoculation with *Fusarium graminearum* and *F. culmorum*, and also decreased DON accumulation in grain [123]. Genome editing tools such as TALENs and CRISPR/Cas9 further allow the precise modification of plant genomes to enhance resistance traits. Among these, CRISPR/Cas9 is particularly favored in crop improvement due to high specificity, efficiency, and cost effectiveness [124]. Additionally, genetically modified maize expressing the anti-insecticidal cry1A(b) gene from *Bacillus thuringiensis* has demonstrated reduced contamination with *Fusarium* mycotoxins, primarily FUM [125]. Despite these advances, the adoption of genetically engineered crops remains controversial, especially within the EU, due to regulatory constraints and ongoing public concern.

An emerging complementary approach within genetic engineering is antibody-mediated technology, which enhances resistance to mycotoxin-producing fungi through the use of recombinant antibodies exhibiting high specificity to fungal antigens [22]. This technology is investigated for controlling *Fusarium*-related diseases and mycotoxin contamination in cereal crops. For instance, a fusion protein consisting of a *Fusarium*-specific recombinant antibody derived from chicken and an antifungal peptide from *Aspergillus giganteus* was expressed in wheat, demonstrating effective protection against *Fusarium* pathogens [126]. Additionally, an antibody–magnetic nanoparticle system was studied as an efficient purification tool for the removal of AFB1 and ZEN from contaminated feed samples, offering a promising alternative to conventional purification methods [127].

### 3.4. Novel Physical Treatments

Cold plasma is increasingly recognized as a promising non-thermal physical method for enhancing food and feed safety owing to its ability to achieve microbial and mycotoxin decontamination without inducing significant alterations in product quality or incurring huge costs. Plasma is defined as a partially or fully ionized gas comprising a mixture of charged particles, free radicals, electrons, and neutral particles. It is typically generated by applying an electric current through neutral gas, resulting in the dissociation of gaseous molecules and the formation of highly reactive oxygen and nitrogen species. These reactive species are responsible for decontamination through interaction with microbial cells and mycotoxins, thereby disrupting their structure and functionality [21,128]. The efficiency of cold plasma treatment is dependent on the molecular structure of the target mycotoxin, as well as processing parameters. For example, the degradation efficiency of DON in wheat has been demonstrated to depend on the plasma voltage, gas type, processing time, matrix characteristics, and moisture content of the food [21,129]. Similarly, the degradation efficiency of T-2 and HT-2 in oat flour is influenced by flour humidity, exposure time, and gas type for cold plasma generation [128]. While current data indicates that cold atmospheric plasma has minimal impact on food composition, further research is needed to fully characterize mycotoxin degradation products, assess their effect on food quality attributes, and optimize application protocols for different food and feed matrices [130].

The potential of microwave and UV treatments in mycotoxin reduction has been recently investigated, as they offer several advantages including low capital cost, ease of integration, and environmental friendliness. Moreover, many food industries already utilize these technologies for other purposes, making their adaptation for mycotoxin control practical and more promising. Microwaves consist of electromagnetic radiation with a wavelength range ranging from 1 mm to 1 m and frequencies between 0.3 and 300 GHz. During microwave treatment, electromagnetic radiation interacts with water molecules and charged ions within the sample, generating heat by friction through processes of dipolar rotation and ion movement. Generated microwave energy can disrupt the chemical bonds of mycotoxins, leading to their degradation [20,131]. Ultraviolet light, covering wavelengths from 100 to 400 nm, is divided into three types: UV-A, long wave (315–400 nm); UV-B, medium wave (280–315 nm); and UV-C, short wave (200–280 nm). UV-C is able to penetrate microbial cells, damaging their DNA, thereby inhibiting DNA replication and transcription, impairing cell function, and leading to cell death [20]. In the context of mycotoxin control, UV radiation is absorbed by the compound, triggering photoreactions that result in mycotoxin degradation [20,131]. Mycotoxin degradation generally increases with increasing UV intensity and irradiation time [20]. For example, a study on AFB1 in peanut oil showed a 95% reduction after 120 s of UV-C (254 nm) exposure, with no observed side effects on oil quality parameters [132]. Similarly, the UV treatment of skim milk samples at 254 nm reduced AFM1 content by up to 50% after 20 min, regardless of the initial contamination level. Factors such as treatment time, depth of samples, and stirring were found to significantly influence the reduction efficiency [133]. In addition, the antimicrobial effect of UV rays depends on the microorganism species and the toxins they produce. For instance, 2 h UV-C treatment (254 nm) of roasted coffee beans was effective in preventing *Aspergillus flavus* contamination (>90%), but was less effective against *A. parasiticus* (<90%) [134]. As the deterioration of certain quality parameters has been observed in different food matrices following UV treatments, further research is needed to optimize its applicability, potentially combining it with complementary techniques [20]. Microwave treatment efficiency also depends on treatment time and power. In the case of the low-power microwave heating of peanuts (360 W for 6 min, 480 W for 5 min, and 600 W for 3 min), AFB1 was reduced by 59–67% [135]. Furthermore, microwaving winter wheat seeds for 15, 30, and 45 s significantly reduced the fungal pathogens *Fusarium* spp. and *Microdochium nivale* (up to 72% and 77%, respectively) [136]. Recent studies applying different microwave powers and UV exposure times under dry conditions to mycotoxin-contaminated rice demonstrated the significant degradation of AFT, AFB1, and OTA content. The highest microwave treatment (720 W, 360 s) achieved the highest toxin reduction, but even lower microwave power levels (560 W, 120 s) resulted in a significant reduction in AFT (33.4%), AFB1 (50.1%), and OTA (75.2%). UV treatments showed optimal reductions at varying exposure times, depending on mycotoxin: AFT 31.1% after 0.5 h, AFB1 44.3% after 4 h, and OTA 57% after 2 h of UV exposure [131].

Other irradiation types have also demonstrated potential in controlling both fungi and mycotoxins in various food and feed matrices. Gamma rays are highly penetrating electromagnetic waves with very short wavelengths (< 0.01 nm), produced by the decay of radioactive isotopes, with high energy enabling organic molecule degradation [20]. During gamma ray irradiation, highly reactive species are generated through reactions with water molecules, including hydrogen radicals, superoxide radicals, and hydroxyl radicals, which can rapidly interact with mycotoxins and facilitate their degradation [20,137]. The effectiveness of gamma irradiation depends on factors such as matrix type, fungal strain and compound, radiation dose, and moisture content. For example, the treatment of sorghum using gamma irradiation at a dose of 3 kGy was found sufficient for eliminating 90% of natural fungal load, while increasing the irradiation dose significantly reduced mycotoxin contamination with a dose of 10 kGy, resulting in 59% and 32% reductions in AFB1 and OTA levels, respectively [138]. Another study showed that the post-storage gamma irradiation of apple juice reduced PAT levels by up to 91% under optimized conditions while still preserving polyphenols [139]. However, gamma irradiation has its limitations. While high doses may negatively impact food quality, as shown in a rice protein study where gamma ray irradiation provided a favorable impact on certain physicochemical and functional properties, it may also compromise its edibility [140]. As gamma rays emit strong radioactivity and therefore pose certain risks, implementing a quality management system and strict control of radiation dose are essential during food treatment [20].

Electron beam irradiation, produced by an electron accelerator with certain energy and intensity, has mechanisms of action comparable to gamma irradiation [20,141]. In maize contaminated with ZEN and OTA, electron beam irradiation at 50 kGy degraded both toxins by about 70%, with kernel moisture and irradiation dosage being important factors affecting efficiency [142]. Moreover, the treatment of red pepper powder with electron beam treatment at 10 kGy for 23 s reduced natural microbiota by 4.5 log CFU/g of total plate counts without altering its physicochemical parameters. Although not effective in direct aflatoxin degradation, indirect mycotoxin control can be achieved through mycotoxigenic fungus inactivation [143].

Pulsed light is also recognized as an effective technique for both microorganism inactivation and mycotoxin degradation, utilizing a combination of photothermal, photochemical, and photophysical effects. This method employs a high-power xenon lamp, powered by a high-voltage direct current source, to deliver intense pulses of broad white light (200–1100 nm). The ultraviolet component of the spectrum contributes to DNA damage in microorganisms, while visible and near-infrared components generate localized surface heating, leading to structural damage and cell death [20]. Pulsed light treatment of barley grains for 15 s at 18.0 J/cm^2^ caused a decrease in *Aspergillus carbonarius* and *A. flavus* counts by 1.2 log and 1.7 log, respectively [144]. A study of pulsed light irradiation on AFB1 and AFB2 contamination in rice and rice bran at a dose of 0.52 J/cm^2^ over 15 s showed 90% and 87% toxin reduction, respectively [145]. Grape juice treatment with pulsed light at 39.0 J/cm^2^ caused OTA degradation of 95%, with no decrease in quality factors such as soluble solids, total organic acids, color, or pH [146]. Although showing significant advantages, pulsed light irradiation has certain efficiency restrictions, particularly when used on dark-colored liquids or uneven surfaces. In addition, the absence of clear regulations regarding optimal flux range poses challenges for its broader application in the food industry [20].

Pulsed electric field is a new type of non-thermal technology that uses short pulses (from nano- to milliseconds) of various electric field strength (0.1–80 kV/cm) to treat food in order to kill microorganisms, reduce mycotoxin levels, and help to extend its shelf life [20,147]. The antimicrobial effect of pulsed electric fields involves the application of intense strong electric fields generated by a pulse power supply, which induces the disruption of microbial cells. The mycotoxin degradation mechanism involves a direct and indirect approach: in the first, high-voltage short-duration electrical pulses interact with mycotoxin molecules, leading to chemical cleavage or redox reaction, while in the second, an electric field alters the physical and biological properties of the mycotoxin’s surrounding matrix, thereby enhancing degradation [20,148]. Pulsed electric treatment has exhibited efficacy in *Aspergillus parasiticus* inactivation and AFT reduction in sesame seeds and red pepper. The maximum microbial inactivation was 60% and 64%, respectively, with 17.28 J of pulsed electric field energy for both matrices, while the decrease in AFB1, AFB2, AFG1, and AFG2 content reached up to 99.99% [149,150]. The degradation potential of pulsed electric fields under two different intensities for 16 common and emerging *Fusarium* and *Alternaria* species was investigated in malting barley. Higher-strength treatment led to higher mycotoxin reduction, with the observed degradation/transformation products of mycotoxins being mostly the result of hydrolysis, elimination, and/or oxidation [151]. The investigation of pulsed electric field on enniatins and beauvericin content in juice and smoothies, at a field strength of 3 kV/cm and specific energy of 500 kJ/kg, revealed mycotoxin degradation between 43% and 70%, along with decreased toxicity of degradation products [152]. Besides its positive effects, pulsed electric field technology also poses several challenges concerning complete pathogen inactivation at lower intensities and high equipment and operational costs; therefore, further research is needed to enable its full industrial application [20].

Collectively, recent advances in mycotoxin mitigation technologies show promising potential by employing diverse and innovative mechanisms. Nevertheless, further research, validation, and optimization will be key to their future implementation [153].

## 4. Conclusions

The ubiquitous nature of mycotoxins underscores the persistent challenge they pose to global food and feed safety and security. Given their well-documented toxicity, health implications, and economic impact, the need for effective mitigation strategies is both urgent and critical. While conventional control methods offer partial solutions, they often face limitations related to efficacy, safety, and impact on product quality. In recent years, certain innovative approaches have gained increasing attention due to their potential to effectively prevent and reduce mycotoxin contamination without leaving toxic residues, and with minimal adverse impacts on nutritional and sensory properties. These emerging strategies include the use of botanicals and phytochemicals, nanotechnology, genetic engineering and antibody-mediated technologies, and non-thermal processing methods. Although promising, to fully realize the benefits of such strategies, further research and upscaling efforts are necessary, together with regulatory frameworks and stakeholder collaboration to safeguard food and feed safety for consumers worldwide.

## Figures and Tables

**Table 1:** Main mycotoxins and their legislative limits for food and feed.

Mycotoxin	Commodity Group	EU Threshold	Legislative Act	US FDA Threshold *
Aflatoxin B1 (AFB1) 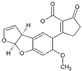	Dried fruits, peanuts, tree nuts, cereals and products, dried spices	2.0–12.0 µg/kg	Commission Regulation (EU) 2023/915 [17]	20.0 µg/kg (total)
	Feed materials, complementary and complete feed	0.005–0.2 mg/kg relative to a feed with a moisture content of 12%	Directive 2002/32/EC [19]	0.02–0.3 mg/kg (total)
Aflatoxin total (B1, B2, G1, G2) (AFT, AFB1, AFB2, AFG1, AFG2)	Dried fruits, peanuts, tree nuts, cereals and products, dried spices	4.0–15.0 µg/kg	Commission Regulation (EU) 2023/915 [17]	
Aflatoxin M1 (AFM1) 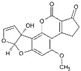	Raw milk, heat-treated milk and milk for the manufacture of milk-based products	0.050 µg/kg	Commission Regulation (EU) 2023/915 [17]	0.5 µg/kg
Ochratoxin A (OTA) 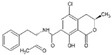	Dried fruits, dried herbs, cereals and products, coffee, dried spices, wine and fruit wine, licorice and products	2.0–80 µg/kg	Commission Regulation (EU) 2023/915 [17]	
	Feed materials (cereals and products) and compound feed for pigs, poultry, cats and dogs	0.01–0.25 mg/kg relative to a feed with a moisture content of 12%	Commission Recommendation (2006/576/EC) [18]	
Patulin (PAT) 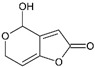	Fruit juices, spirit drinks, cider and other fermented products derived from apples, solid apple products	25–50 µg/kg	Commission Regulation (EU) 2023/915 [17]	50 µg/kg
Deoxynivalenol (DON) 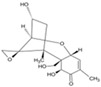	Unprocessed cereals, cereal milling products, bakery products, pasta	250–1750 µg/kg	Commission Regulation (EU) 2023/915 [17]	1000 µg/kg
	Feed materials (cereals and products, maize by-products), compound feed	0.9–12 mg/kg relative to a feed with a moisture content of 12%	Commission Recommendation (2006/576/EC) [18]	4–30 mg/kg
Zearalenone (ZEN) 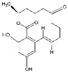	Unprocessed cereals, milling products, bakery products, refined maize oil	50–400 µg/kg	Commission Regulation (EU) 2023/915 [17]	
	Feed materials (cereals and products, maize by-products), compound feed for piglets, gilts, puppies, kittens, dogs and cats, sows and fattening pigs, calves, dairy cattle, sheep and goats	0.1–3 mg/kg relative to a feed with a moisture content of 12%	Commission Recommendation (2006/576/EC) [18]	
Fumonisin sum (B1, B2) (FUM, FB1, FB2) 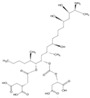	Unprocessed maize, maize milling products, maize-based products	800–4000 µg/kg	Commission Regulation (EU) 2023/915 [17]	2000–4000 µg/kg (sum of B1, B2, B3)
	Feed materials (maize and products) and compound feed for pigs, horses, fish, poultry, calves, lams, ruminants	5–60 mg/kg relative to a feed with a moisture content of 12%	Commission Recommendation (2006/576/EC) [18]	5–60 mg/kg (sum of B1, B2, B3)
Citrinin (CIT) 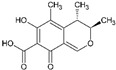	Food supplements based on rice fermented with red yeast *Monascus purpureus*	100 µg/kg	Commission Regulation (EU) 2023/915 [17]	
Ergot alkaloids (EAs) 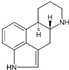	Cereal milling products	50–100 µg/kg	Commission Regulation (EU) 2023/915 [17]	
T-2 and HT-2 toxin (T-2, HT-2) 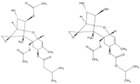	Unprocessed cereals, cereals for human consumption, cereal milling products, cereal bran, bakery products, pasta, cereal snacks, oat flakes, breakfast cereals	20–1250 µg/kg	Commission Regulation (EU) 2023/915 [17]	
	Compound feed for cats	0.05 mg/kg relative to a feed with a moisture content of 12%	Commission Recommendation (2006/576/EC) [18]	
	Oat milling products and other cereal products, compound feed with the exception of feed for cats	250–2000 µg/kg relative to a feed with a moisture content of 12%	Commission Recommendation (2013/165/EU) [24]	

* https://www.fda.gov (accessed on 2 October 2025).

## Data Availability

No new data were created or analyzed in this study.
